# Perianal condylomata acuminata in pregnancy

**DOI:** 10.11604/pamj.2021.38.172.28077

**Published:** 2021-02-16

**Authors:** Damaskini Polychroni, Asterios Nidimos

**Affiliations:** 1 Department of Obstetrics and Gynecology, General Hospital of Mytilene, Lesvos 81132, Greece

**Keywords:** Condyloma acuminatum, perianal warts, pregnancy, human papilloma virus

## Image in medicine

A 23-year-old woman of low socioeconomic status presented to our obstetric department reporting pain in the perineal area, occasional bleeding during defecation and discomfort at the sitting position. The woman was nulliparous, at the 34^th^ week of gestation and had an unattended pregnancy, since she had never visited a healthcare provider the last three years. Physical examination revealed multiple, irregular vegetative lesions covering the perianal region. These cauliflower-like masses where hard and painless with maximum diameter 10cm, while vagina and vulva were free of warts. The patient was scheduled for elective caesarean section at the 38^th^ week of pregnancy, because large warts were blocking the natural birth canal. She was referred to a tertiary health care unit for further counselling and treatment after postpartum period.

**Figure 1:**
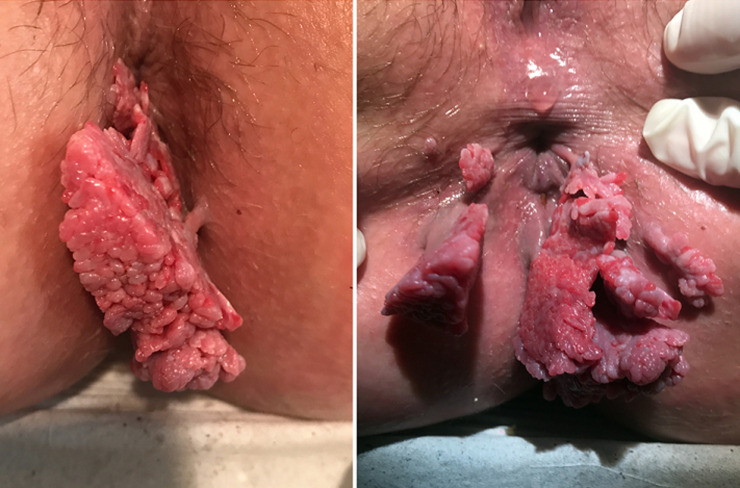
large perianal condylomata acuminata in pregnancy

